# Community-Based Participatory Research to Improve Cardiovascular Health Among US Racial and Ethnic Minority Groups

**DOI:** 10.1007/s40471-022-00298-5

**Published:** 2022-07-11

**Authors:** Norrisa Haynes, Amanpreet Kaur, JaBaris Swain, Joshua J. Joseph, LaPrincess C. Brewer

**Affiliations:** 1 Division of Cardiology, University of Pennsylvania, Philadelphia, PA USA; 2 The University of Pennsylvania, Leonard Davis Institute of Health Economics, Philadelphia, PA USA; 3 Biotech Commons, University of Pennsylvania Libraries, Philadelphia, PA USA; 4 University of Pennsylvania Center for Public Health Initiatives, Philadelphia, PA USA; 5 Division of Cardiovascular Surgery, University of Pennsylvania, Philadelphia, PA USA; 6 Division of Endocrinology, Diabetes and Metabolism, The Ohio State University Wexner Medical Center, Columbus, OH USA; 7 Department of Cardiovascular Medicine, Division of Preventive Cardiology, Mayo Clinic College of Medicine, Rochester, MN USA; 8 Center for Health Equity and Community Engagement Research, Mayo Clinic, Rochester, MN USA

**Keywords:** Community-based participatory research, Community-engaged research, Social determinants of health, Cardiovascular disease, Cardiovascular health, Racial and ethnic minority groups

## Abstract

**Purpose of Review:**

This review aims to assess the contemporary community-based participatory research (CBPR) literature seeking to improve the cardiovascular health of racial and ethnic minority groups in the USA with a higher burden of cardiovascular risk factors and social determinants of health. It summarizes recent CBPR studies based on the American Heart Association Life’s Simple 7 (LS7) framework, delineating seven modifiable health behaviors and clinical factors to promote cardiovascular health.

**Recent Findings:**

Although limited in quantity, studies demonstrated preliminary effectiveness in improving individual and a composite of LS7 indicators by employing strategies centered around fortifying social networks, integrating group activities, leveraging technology, incorporating faith-based and spiritual practices, and implementing changes to the built environment.

**Summary:**

Future directions for investigators engaged in CBPR include building on the existing body of evidence through more comprehensive studies, scaling effective interventions, and translating CBPR findings to influence health policy to better address health disparities.

## Introduction

Cardiovascular (CV) disease, as defined by the American Heart Association (AHA), includes hypertension (HTN), peripheral vascular disease, coronary heart disease, stroke, and heart failure [1]. Specific racial and ethnic minority groups in the USA, including African American (AA), LatinX, and American Indian/Alaska Native (AI/AN) individuals, have a disproportionately higher burden of CV risk factors and deleterious social determinants of health (SDoH) compared to White Americans [1, 2]. The AHA compiled seven core health behaviors and clinical factors contributing significantly to cardiovascular health (CVH): blood pressure (BP), cholesterol, glycemia, body mass index (BMI), physical activity (PA), smoking, and diet [3]. The LS7 is objectively measured via an evidence-based score metric of CVH ranging from poor to ideal [4]. The American Heart Association Life’s Simple 7 LS7 scoring provides an objective means for researchers, healthcare professionals, and policymakers to identify, monitor, and address CVH disparities [2, 4].

Many LS7 health behaviors and clinical factors are impacted by social determinants of health (SDoH). SDoH are the economic, social, environmental, and psychosocial conditions in which individuals are born, grow, live, work, and age [5, 6]. These conditions are shaped by the distribution of resources and power at local and national levels [5]. Studies indicate that SDoH has a higher impact on population health than traditional medical care alone [7]. Additionally, socioeconomic and racial/ethnic disparities in cardiovascular risk factors and mortality persist despite skyrocketing national healthcare expenditures, which have tripled over the past 20 years to $4.1 trillion/year in 2020 [8]. Thus, although evidence demonstrates longstanding racial/ethnic and socioeconomic disparities in CVH, interventions at the population level have lagged [9]. The solutions to address these disparities include prevention, community engagement, and policy change. Community-based participatory research (CBPR) attempts to address these glaring disparities by engaging communities in the research development, design, and implementation lifecycle, thereby addressing the social, cultural, and environmental contexts of communities. Additionally, CBPR commences a process of reckoning with medical mistrust in research and clinical care [10]. It can build evidence through actionable research to bridge the gap between academia, surrounding communities, and local policymakers.

### What Is CBPR?

CBPR is focused on developing collaborative partnerships facilitating equal input from the community and its stakeholders throughout the research process, including planning, implementation, evaluation, and dissemination [11, 12]. CBPR builds on several approaches, including participatory and action research [13]. CBPR exists within the broader field of community-engaged research, which is a spectrum that spans from community-placed research on one end to CBPR and community-driven research on the other [13]. Community-placed research describes research conducted in the community; however, community members have no control over the research agenda. All of the control is retained by the academic institution. However, CBPR is genuine “of, by, and for the people” as community members have equal decision-making power and ensure that the primary goal of the research is to benefit the community [11, 12].

### History of CBPR

CBPR was created to address health disparities and promote community empowerment while applying scientific principles and rigor [14]. It falls within participatory research, focusing on continuous inquiry, evaluation, and action implemented *with* instead of *on* marginalized individuals [15]. The origins of CBPR were built on participatory action research popularized during the civil rights and liberation movements in the US and Latin America within disenfranchised communities [16]. CBPR embraces the concept that community members should be better positioned as equal partners in inquiry instead of “empty vessels and objects of inquiry” [15]. It also implies relationship-building and trust among academic and community partners by disrupting the perceived “us versus them” construct of traditional research approaches [17].

Historical improprieties in medical research from unethical research practices (e.g., Tuskegee Syphilis Study and Henrietta Lacks cell line use) undoubtedly played a role in the evolution of CBPR in the USA [18]. The long history of medical and scientific exploitation understandably has led to deep-seated mistrust [19]. Through CBPR, academics have an opportunity to address community concerns, including historic misgivings, to create sustainable solutions for change in historically marginalized and socioeconomically disadvantaged communities [19].

### CBPR Principles and Approaches

CBPR is a collaborative approach to research that can incorporate various research designs, including experimental, nonexperimental, case studies, longitudinal, ecological, and implementation science designs. Additionally, CBPR data collection methods often include both qualitative and quantitative analyses with the engagement of community members in each step of the process [20].

CBPR frameworks typically incorporate five formative stages: (1) building partnerships, (2) developing rules of operation and decision-making, (3) study selection and design, (4) data analysis, and (5) dissemination and translation of research findings into policy and practice [20, 21]. To forge academic and community partnerships, academics must combine their research skills with humility, respect for the community, patience, and the will to build community capacity. Additionally, the formation of a steering committee or advisory board made up of multidisciplinary community representatives and stakeholders is critical to the success of the community partnership. For example, the Fostering African-American Improvement in Total Health! (FAITH!) program, a CBPR initiative in Minnesota, outlines the importance of creating a community steering committee (CSC) to understand nuanced insights on local contexts and engage community members in research and disseminate research findings to the community [22]. Core CBPR principles and corresponding best practices are listed in Table 1.

**Table 1:** CBPR principles and best practices

CBPR principles	Best practices
1. Collaborative and equitable partnerships in all research phases, which involves a power-sharing process	Establish community steering committees/advisory boards, bylaws, and a decision-making framework
2. Recognize community as a unit of identity	Acknowledge, respect, and embrace factors and interests that connect community members (e.g., traditions, norms, values, language, customs, and goals)
3. Build on strengths and resources within the community	Leverage community partner and stakeholder ability to engage with communities in health promotion and community initiatives
4. Facilitate co-learning and capacity building among all partners	Academic partners learn a community’s history, culture and broader social context, and community partners learn research process methodologies and grant writing
5. Focus on problems relevant to the community using an ecological approach	Incorporate community priorities by allowing community members to generate research questions and hypotheses
6. Balance research and action for the mutual benefit of all partners	Allow community members to generate intervention ideas and guide recruitment, retention, and implementation strategies
7. Disseminate findings and knowledge to the broader community and involve all partners in the dissemination process	Community partners should participate in data interpretation and co-author publications. Data should be shared with prioritized communities via community-centric means (e.g., town halls, newsletters, video summaries, social media, etc.)
8. Promote a long-term process and commitment to sustainability	Embed sustainability plans into grant applications, advocate for policy change, and establish partnerships built on trust, respect, and friendship

This review aims to evaluate contemporary CBPR intervention studies seeking to improve the CVH of racial and ethnic minority groups in the USA with a high burden of CV risk factors and SDoH. We also identify gaps in the contemporary CBPR body of evidence and highlight future directions for CBPR researchers to achieve CVH equity.

## Methods

In February 2022, relevant peer-reviewed literature was identified on PubMed, CINAHL, and social work abstracts to identify articles from 2019 onwards to evaluate the contemporary literature published over the past 3 years. Each search strategy was customized to work within a specific database. See Table 2 below for the search strategies along with the filters used. Additionally, relevant foundational and background literature published before 2019 was manually identified through citation chasing. Studies published before 2019 were included only if their findings were important and if the findings were scarcely available in the literature. Given the small volume of studies, both complete and proposed CBPR studies were accepted. Citation chasing was a critical method to identify examples of CBPR within racial and ethnic minority groups, in which there was limited research published within the past three years. This review focused primarily on US-born, non-immigrant populations, particularly AAs.

**Table 2:** Search strategies and filters by database

Database	Search strategy	Publication date range	Geography filter	Number of results
PubMed	(“Community-based participatory research” [Mesh] OR “community-based participatory research” OR CBPR) AND (heart OR cardiovascular) AND (“USA” OR American)	2019 to 2022		52
Social Work Abstracts	(“Community-based participatory research” or CBPR) AND (cardiovascular or health)	2019 to Current		3
CINAHL	Search 1: (“community-based participatory research” OR CBPR) Search 2: (heart OR cardiovascular) Search 3: (“USA” OR American) Search 4: (improve OR increase OR enhance OR promote) Search 5: S1 AND S2 AND S3 AND S4	2019 to 2022	USA	148

### Study Screening and Selection

The combined results of three databases were manually screened due to irrelevant titles and abstracts (i.e., non-US study location; not focused on adult population; not focused on CVH, diabetes, blood pressure, diet, PA, obesity, or other factors of interest). In addition, only papers available in English were included.

### Summary of Search Results

The 203 results were combined into a shared EndNote folder. After deduplicating with EndNote, there were 196 results. The manual screening was performed, and the inclusion criteria were as follows: CBPR approach, adult study population, racial/ethnic groups of interest, and cardiovascular health intervention study design.

Forty articles were included in total, one of which was a systematic review which was included in multiple categories: 5 articles focused on BP, 1 on cholesterol, 4 on glycemia, 5 on BMI, 10 on PA, 5 on smoking cessation, 6 on diet, and 4 on all LS7 clinical factors and behaviors. There was a primary focus on intervention studies through quasi-experimental and randomized controlled trial (RCT) designs; however, ecological assessments and qualitative studies informing future intervention studies were also included.

### Review of Studies Using the AHA Life Simple 7 Framework

Cardiovascular health clinical factors:

#### BP

##### AA Communities

A systematic review of peer-reviewed literature on CBPR aimed at improving one or more LS7 factors among AAs identified intervention strategies that successfully improved BP in AA communities. The interventions focused on enhancing PA and dietary change, with an average decrease in systolic BP of 8 mmHg following the interventions [23••]. The most common PA intervention included coach-led walking groups. Faith-based interventions also showed effectiveness in reducing BP [23••]. For changes in diet, emphasis on the DASH diet, the establishment of community gardens, and healthy food shopping assistance programs were the most commonly effective interventions [23••]. Along the lines of instituting environmental changes to address BP indirectly, one nurse-driven environmental justice CBPR project found that lead levels were higher than the health limits in 10.4% of drinking water samples in an AA community [24]. This situation is significant because lead exposure is associated with increased BP and risk of HTN [25].

There are two community-engaged trials worth mentioning due to their significant reduction in BP at the community level. The pharmacist-led BP control study in Black barbershops and the HTN trial of therapeutic lifestyle change in Black churches achieved a 27 and 6 mmHg decrease in systolic BP, respectively [26, 27]. Although these trials are not CBPR studies, they prove that well-funded and rigorously conducted community-engaged research can significantly impact CVH.

##### LatinX Communities

An RCT randomized 98 Mexican American adult participants to a promotora (community health worker) HTN education intervention or a control arm with language-appropriate educational materials. There was no statistically significant change in the primary outcome of BP at 9 weeks post-intervention. However, participants in the intervention group reported a statistically significant improvement in dietary salt/sodium intake (*p* = 0.03) [28].

##### AI/AN Communities

One RCT among Pacific Northwest tribes in the US plans to randomly assign 135 at-risk AI/AN adults to a CV disease prevention intervention or a comparison arm [29]. The CV disease prevention arm encourages regular exercise and healthy eating through traditional culturally tailored motivational interviewing and personal coaching performed by professionals vetted by a board of community and academic members. Most coaches and motivational interviewers are AI/AN [29]. BP is one of the primary outcomes [29]. The results of this study have not yet been published.

#### Cholesterol

According to a systematic review, there was an average increase in mean HDL of 5.7 mg/dL and a reduction in mean total cholesterol of 2.2 mg/dL among AAs through programs focused on PA and dietary changes [23••]. More CBPR ecological assessment studies are needed to determine community-level ASCVD risk and current statin utilization in racial and ethnic minority groups. Additionally, CBPR statin intervention trials could significantly help to address hyperlipidemia in racial and ethnic minority communities.

#### Glycemia

##### AA Communities

Several strategies are effective in decreasing blood glucose and hemoglobin A1c [23••]. These strategies include trained coaching to deliver culturally tailored curricula focused on self-care, walking groups, and diet and diabetes education [23••]. Through these strategies, there was an average decrease in blood glucose of 6.4 mg/dL and a decrease in hemoglobin A1c of 0.7% [23••].

##### AI/AN Communities

One study among Marshallese adults with type 2 diabetes incorporated family diabetes self-management education, which involved the engagement of family members of participants with diabetes in education and diabetes management. The study showed increased engagement in glucose monitoring and outpatient healthcare provider follow-up among participants [30]. Social support and cultural factors can fortify social support networks, reinforce cultural identity, and enhance diabetes self-management [31]. One study that created and disseminated a glucose monitoring video in Marshallese with English subtitles achieved a 1.45% reduction in hemoglobin A1c (*p* = 0.006) [32].

#### BMI

##### AA Communities

Many studies focusing on improving BMI or obesity promoted wellness plans that target PA and diet to achieve weight loss goals. Most studies utilized group activities, with a few incorporating individualized meetings to discuss weight management [23••]. For example, the faith influencing transformation (FIT) study was an RCT of an 8-month weight loss intervention in AA churches [33]. The intervention included self-help materials, YMCA-facilitated weekly group weight loss classes, church activities (sermons and responsive readings), and church-community text/voice messages to promote healthy eating and PA. Overall, there was a positive trend toward weight loss in the intervention group, although not statistically significant [33]. Another study among a cohort of AA women with overweight/obesity status demonstrated that those with lower body image dissatisfaction had greater dietary self-regulation to reduce fat and caloric intake compared to those with higher body image dissatisfaction. Thus, psychosocial factors such as body image perception are potential culturally relevant lifestyle intervention targets among AA women [34].

##### LatinX Communities

A cluster RCT in South Los Angeles that included two large LatinX churches (~ 20,000 parishioners) and two mid-sized AA Baptist churches (~ 200 parishioners) noted significant changes in BMI [35]. The intervention included health promotion sermons and community mapping of food and physical environments. The intervention resulted in a statistically significant decrease in BMI among participants at 5 months post-intervention (− ∆0.08 kg/m^2^, *p* < 0.05) [36].

Cardiovascular health behaviors:

#### PA

##### AA Communities

Successful PA interventions incorporated educational materials to increase at-home activity levels and develop weekly fitness goals [23••]. Evidence-based interventions such as PREMIER, a behavior change intervention focused on goal setting for diet, PA, and alcohol consumption, were utilized in rural AA communities [37]. Many successful frameworks involved setting fitness goals and PA plans with larger groups facilitated by coaches or leaders from the community to better serve and represent the population [23••]. Other studies included supervised exercise ranging from 10 to 90 min of weekly group exercise. The most effective interventions utilized several approaches, including goal setting and individual PAs such as home exercises and group classes [23••]. Mobile health applications have also proven to be very effective in increasing PA in AA communities [38, 39••, 40].

It is also known that community infrastructure and economic environments correlate with PA [41]. For example, higher-income communities are more likely to have recreational facilities and better sidewalk conditions that link with increased PA [41]. A study by Moore et al. sought to investigate resilience strategies and correlation with PA in a low-income urban AA community [41]. They found that prosocial behavior (behavior that benefits another person or the community) played a significant role in promoting regular PA [41].

##### LatinX Communities

Based on a focus group analysis that utilized a CBPR approach, Latinx residents in a Midwestern city revealed not feeling comfortable engaging in PA alone [42]. In addition, respondents expressed not knowing any family or friends who liked to exercise, not having anyone to exercise with in the area, and not feeling comfortable being outside due to safety concerns [42].

##### AI/AN Communities

The Incorporation of Original Instructions (OI) and Indigenous Knowledge (IK), cultural practices among AI/AN and Native Hawaiian groups, have the potential to improve CVH behaviors such as PA [43, 44]. OI is defined as ancient teachings regarding practices and responsibilities that enact IK and are expressed through stories, songs/chants, dances, ceremonies, and calendrical spiritual teachings and governance systems. IK incorporates perceptions, decision-making processes and provides the context of underlying values, ethics, and responsibilities [43]. Examples include embracing Native language, arts, music, dance, and history. For the Chocktaw tribe, this includes ancestral connectedness and knowledge gained by re-walking ancestors' footsteps along the trail of tears in consultation with the community and cultural leaders [43, 44]. Qualitative data from tribal members suggest that the physical and emotional challenge of the ancestral trail promoted changes in health behaviors, attitudes, and beliefs [44]. The role of social support in PA was also highlighted among AI tribes in Oklahoma. Based on a survey-based study, participants who exercised with others or pets were significantly more likely to achieve regular, consistent PA than those who exercised alone [45].

#### Smoking

##### AA Communities

Successful interventions conducted in predominantly AA communities integrated similar methods, including biblical scripture messaging in the church setting, nicotine replacement therapy, and counseling. Interventions that incorporated free nicotine replacement therapy sustained the most extended abstinence rates [23••]. The average post-intervention self-reported quit rate was ~ 24% [23••]. In one specific study, counseling interventions facilitated by trained peers at community locations with monetary and non-monetary (points) incentives increased participation [46]. The study was successful given that 21% of participants quit smoking and there was a 52% retention rate (defined as attending at least half of the counseling sessions).

##### AI/AN Communities

The All Nations Breath of Life smoking cessation pilot study was tested in urban and reservation communities from the southern and northern plains. The program utilized weekly in-person support group sessions, phone calls, and motivational interviewing. Sessions focused on smoking cessation and health, which included culturally relevant content. Preliminary results showed a participant self-reported quit rate of 65% at program completion and 25% maintained cessation at 6 months [47].

Smoking prevalence rates among pregnant AI and AN women are high at 36% and 21%, respectively. A pilot RCT intervention randomized pregnant AN women to either the intervention arm, which consisted of counseling and videos of women discussing how they quit using positive cultural activities, or the control arm of brief 5-min counseling at prenatal visits [48]. Despite rating the intervention as highly acceptable, participation was low. Additionally, biochemically verified abstinence rates were suboptimal, with 0% in the intervention and 6% in control. More qualitative studies to enhance outreach and improve the interventions’ efficacy are needed in this population [48].

#### Diet

##### AA Communities

Interventions targeting dietary changes commonly focus on increasing the intake of fruits, vegetables, whole grains, and fiber while decreasing sugar and fat consumption. [23••] Portion control and healthy snacking habits were also commonly emphasized [23••]. Among successful interventions, the average increase in fruit and vegetable intake was 0.7 servings/day [23••]. Additional strategies included live demonstrations, cooking classes, and taste testing [23••]. Church partnerships incorporating Bible study and small group-based nutrition education provided by pastors and church members also increased vegetable uptake [49]. Faith-based dietary coaching to reduce calories, fat, and salt were also effective [50]. One study showed that church-based interventions could have long-lasting effects, up to 24 months [51]. Other successful approaches included improving access to farmer's markets and community gardens and directly providing produce or money to purchase healthy food [23••].

##### LatinX Communities

Tu Salud ¡Sí Cuenta!, a quasi-experimental study, investigated the impact of a community-wide campaign on eating behaviors among Mexicans in America [52]. The main outcome measures were healthy and unhealthy eating indices. The campaign involved disseminating culturally and language-appropriate messages on PA and eating behaviors transmitted via TV, radio segments, newsletters, and community health workers. Compared to the control group, there were significantly lower rates of unhealthy eating in the intervention group [52].

##### AI/AN Communities

In some AI/AN communities, food deserts are pervasive. CBPR interventions have prioritized tribal food environments, focusing specifically on tribal-owned convenience stores [53]. One such study randomized eight tribal convenience stores. It implemented “healthy makeovers” within the stores, which involved increasing the availability of healthy and nutritious foods, reducing the pricing for healthy foods, and aggressively marketing healthy foods. The goal was to study the effectiveness of such interventions, including their cost-effectiveness, with the goal of scaling-up future endeavors [53]. The intervention resulted in the purchasing of healthier food items [53, 54].

#### All LS7 Components

Few studies have addressed all seven of the LS7 clinical factors and behaviors. One community-engaged qualitative study sought to study perceived barriers and facilitators to optimal CVH among AA women living in public housing [55]. The focus groups revealed that stress and finances were primary barriers. Limited access to affordable healthy food was also identified as a barrier while social support was identified as a primary facilitator [55].

Another study focused on improving CVH in Black men through a 24-week community-based team lifestyle change intervention [56••]. The intervention included sessions focused on cooking, grocery shopping, mental health, historical trauma, stress, financial wellness, and cancer screening (issues that mattered to the community) [56••]. There was a statistically significant increase in the mean LS7 composite score from 7.12 (intermediate-range) to 8.05 (ideal range) at post-intervention among the participants (+ ∆0.93 points, *p* < 0.0001) [56••].

The FAITH! Program studies utilized in-person and mobile health-based intervention studies to improve LS7 behaviors and clinical factors among AA faith-based communities in Minnesota [57, 58]. The FAITH! App was co-created with community members and included educational multimedia modules with interactive diet/PA self-monitoring and social networking through discussions and sharing boards. The 10-week intervention resulted in 6 mmHg reductions in both systolic and diastolic BP (*p* < 0.01) among participants. Regarding behaviors, fruit/vegetable servings/day increased from 3.4 to 4.5 servings/day (*p* < 0.001); moderate-intensity PA rose from 35 to 75 min/week (*p* = 0.04) at 28-weeks post-intervention. Lastly, the mean LS7 composite score increased from 8.3 to 9.0 (*p* = 0.05) [39••].

## Discussion

**Fig. 1:**
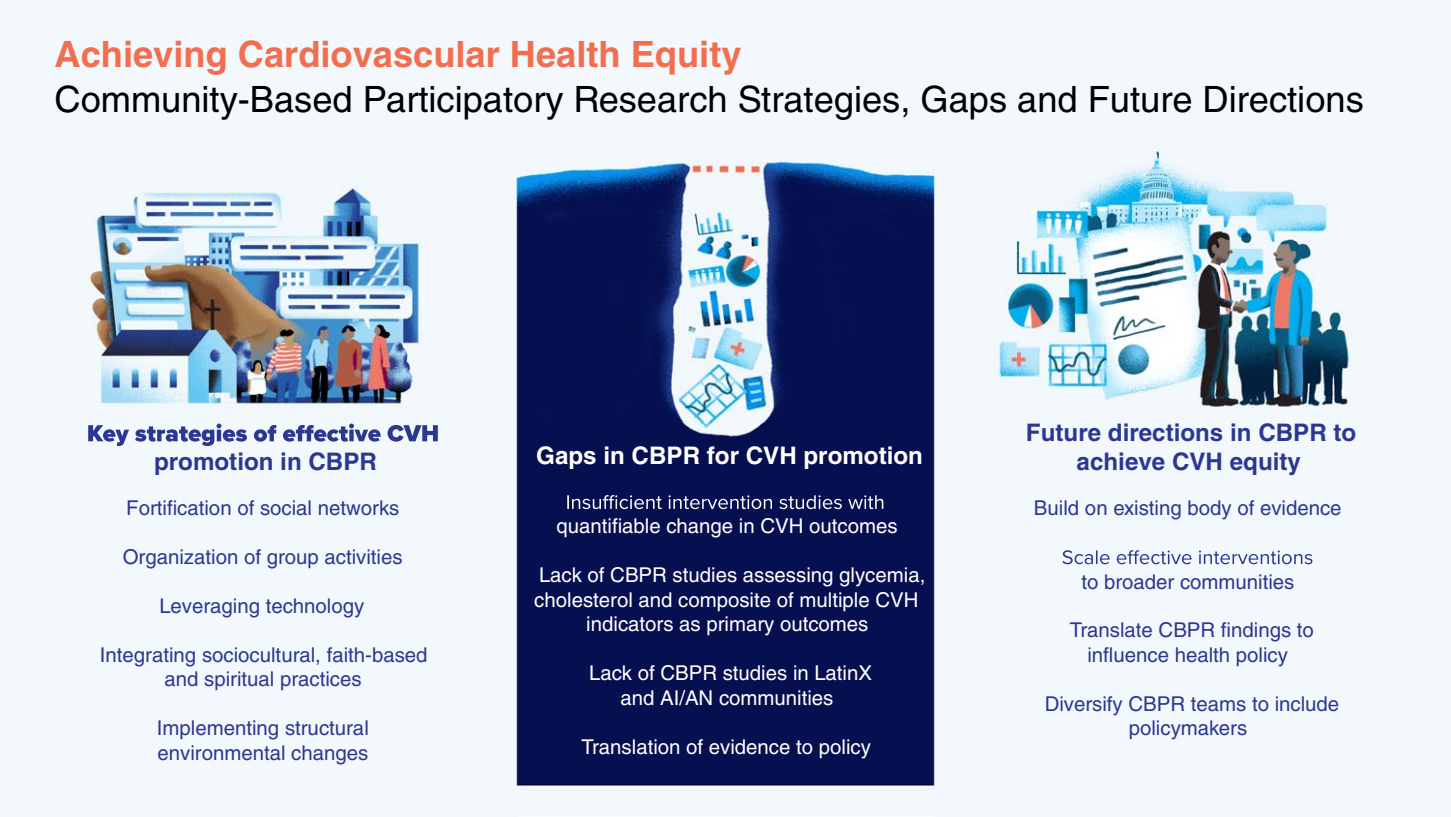
Achieving cardiovascular health equity: community-based participatory research strategies, gaps, and future directions. Synthesis of contemporary CBPR literature revealed key strategies of effective CVH promotion, gaps in study designs (types, outcomes, and populations prioritized), and translation to policy, as well as opportunities for future directions to achieve CVH equity. *AI/AN*, American Indian/Alaska Native; *CBPR*, community-based participatory research; *CVH*, cardiovascular health

This review provides a summary of contemporary peer-reviewed literature to support CBPR approaches for the promotion of ideal CVH behaviors and clinical factors among racial and ethnic minority groups in the USA. By considering the SDoH of specific communities, CBPR has the unique ability to address CV disease disparities by creating and delivering tailored community interventions through buy-in and support from key community stakeholders. Based on our review, several effective strategies for CVH promotion emerged. These key strategies included fortifying social networks, organizing group activities, leveraging technology, accepting faith-based and spiritual practices, and implementing structural environmental changes (Fig. 1). CBPR studies conducted in predominantly AA communities relied heavily on churches to implement interventions as influential social networks/structures to incite collective behavioral change. For studies in predominantly LatinX communities, the utilization of community health workers, family participation, and appropriate language were essential factors. Significant factors incorporated into AI/AN community studies included spirituality, language, traditional customs such as dance, and an acceptance of core cultural virtues endowed by ancestors and elders. However, there remains a lack of CBPR intervention studies simultaneously targeting both individual (e.g., cholesterol) and multiple LS7 factors. These are particularly lacking in the LatinX and AI/AN communities. Additionally, policy change was not a central focus of the studies included in this review.

### Key Recommendations/Future Directions

While some CBPR studies have shown preliminary effectiveness in improving CVH among specific racial/ethnic minority groups, interventions focused on policy change are direly needed. However, bridging the gap between the growing body of CBPR evidence and policy is challenging. Most CBPR policy partnerships have focused on environmental justice and occupational health [59]. Communities predominantly occupied by racial/ethnic minority groups are more likely to be socioeconomically disenfranchised with poor quality housing, lack of green spaces, high crime, and food deserts [59–61]. There are difficulties in moving evidence from the sphere of academia into policy due to the fundamental disconnect between researchers and policymakers [59]. Policymakers are more likely to use evidence applicable to their policy agendas [59]. Bridging this divide requires researchers and policymakers to step out of their siloes to create CBPR interventions together with policy change for the benefit of underserved communities as the primary focus. This translates to including policymakers on CBPR teams, advocating for social and political change, engaging with the media, and communicating CBPR evidence in the lay press and policy briefs (Fig. 1). Widespread adoption of these strategies may be effective in catalyzing necessary policy change [59].

## Conclusion

This review of contemporary CBPR studies provides preliminary evidence to support this approach in improving CVH behaviors and risk factors among US racial and ethnic minority groups with a high burden of CV risk factors and SDoH. Potentially effective CBPR strategies may leverage social networks, group activities, technology, faith-based and spiritual practices, and structural environmental changes. Future directions for investigators engaged in CBPR should include building on the existing body of evidence through more prominent, encompassing studies to demonstrate efficacy for broader scalability. Such studies will foster the translation of findings to influence health policy to better address health disparities and ultimately achieve health equity. Substantial support from major funding agencies is crucial to expand CBPR-informed interventions and solutions among racial and ethnic minority groups within socioeconomically disadvantaged communities.

## Abbreviations

AA: African American
AHA: American Heart Association
AI/AN: American Indian/Alaska Native
BMI: Body mass index
BP: Blood pressure
CBPR: Community-based participatory research
CSC: Community Steering Committee
CV: Cardiovascular
CVD: Cardiovascular disease
CVH: Cardiovascular health
FAITH: Fostering African-American Improvement in Total Health
IK: Indigenous Knowledge
LS7: Life’s Simple 7
OI: Original Instructions
PA: Physical activity
RCT: Randomized controlled trial
SdoH: Social determinants of health

## References

[1] Tsao CW, Aday AW, Almarzooq ZI, Alonso A, Beaton AZ, Bittencourt MS (2022). Heart disease and stroke statistics-2022 update: a report from the American Heart Association. Circulation.

[2] Key Facts on Health and Health Care by Race and Ethnicity | KFF [Internet]. [cited 2022 Mar 29]. Available from: https://www.kff.org/racial-equity-and-health-policy/report/key-facts-on-health-and-health-care-by-race-and-ethnicity/

[3] Lloyd-Jones DM, Hong Y, Labarthe D, Mozaffarian D, Appel LJ, Van Horn L (2010). Defining and setting national goals for cardiovascular health promotion and disease reduction: the American Heart Association’s strategic impact goal through 2020 and beyond. Circulation.

[4] Mok Y, Sang Y, Ballew SH, Rebholz CM, Rosamond WD, Heiss G, et al. American heart association's Life's Simple Seven at middle age and prognosis after myocardial infarction in later life. J Am Heart Assoc. 2018;7(4). 10.1161/JAHA.117.007658 PMC5850193 29455158

[5] Social determinants of health [Internet]. [cited 2022 April 14]. Available from: https://www.who.int/health-topics/social-determinants-of-health

[6] Powell-Wiley TM, Baumer Y, Baah FO, Baez AS, Farmer N, Mahlobo CT (2022). Social determinants of cardiovascular disease. Circ Res.

[7] HealthPartners Institute, Magnan S. Social determinants of health 101 for health care: five plus five. NAM Perspectives. 2017;7(10).

[8] How has U.S. spending on healthcare changed over time? - Peterson-KFF Health System Tracker [Internet]. [cited 2022 Feb 6]. Available from: https://www.healthsystemtracker.org/chart-collection/u-s-spending-healthcare-changed-time/

[9] Joseph JJ, Ortiz R, Acharya T, Golden SH, López L, Deedwania P (2021). Cardiovascular impact of race and ethnicity in patients with diabetes and obesity: JACC focus seminar 2/9. J Am Coll Cardiol.

[10] Gray Ii DM, Nolan TS, Bignall ONR, Gregory J, Joseph JJ (2022). Reckoning with our trustworthiness, leveraging community engagement. Popul Health Manag.

[11] Hollen ML, Balcazar H, Medina A, Ahmed N (2002). The North Texas Salud Para Su Corazón (Heart for your Health) outreach initiative: serving Hispanics in Forth Worth and Dallas. Texas Public Health Assoc J.

[12] Health Promotion in Multicultural Populations - Google Books [Internet]. [cited 2022 Feb 22]. Available from: https://www.google.com/books/edition/Health_Promotion_in_Multicultural_Popula/heEgAQAAQBAJ?hl=en&gbpv=1&dq=Castro+F+Balcazar+H+Cultural+considerations+in+prevention+practice+2000+northern+California+conference+on+prevention+technology+San+Jose,+CA+&pg=PA222&printsec=frontcover

[13] Ward M, Schulz AJ, Israel BA, Rice K, Martenies SE, Markarian E (2018). A conceptual framework for evaluating health equity promotion within community-based participatory research partnerships. Eval Program Plann.

[14] Wallerstein NB, Duran B (2006). Using community-based participatory research to address health disparities. Health Promot Pract.

[15] Macaulay AC (2017). Participatory research: what is the history? Has the purpose changed?. Fam Pract.

[16] Nelson A. Body and soul: the black panther party and the fight against medical discrimination. University of Minnesota Press; 2011.

[17] Why are health disparities everyone’s problem? | Hopkins Press [Internet]. [cited 2022 Apr 14]. Available from: https://www.press.jhu.edu/books/title/12659/why-are-health-disparities-everyones-problem

[18] Drame ER, Irby DJ (2016). Black participatory research.

[19] Skinner HG, Clancy L, Vu MB, Garcia B, DeMarco M, Patterson C (2015). Using community-based participatory research principles to develop more understandable recruitment and informed consent documents in genomic research. PLoS ONE.

[20] Horowitz CR, Robinson M, Seifer S (2009). Community-based participatory research from the margin to the mainstream: are researchers prepared?. Circulation.

[21] Community-Based Participatory Research Principles | Detroit Urban Research Center [Internet]. [cited 2022 Mar 14]. Available from: https://detroiturc.org/about-cbpr/community-based-participatory-research-principles

[22] Manjunath C, Ifelayo O, Jones C, Washington M, Shandling S, Williams J, et al. Addressing cardiovascular health disparities in Minnesota: establishment of a community steering committee by FAITH! (Fostering African-American Improvement in Total Health). Int J Environ Res Public Health. 2019;16(21). 10.3390/ijerph16214144 PMC6862476 31661826

[23] Elgazzar R, Nolan TS, Joseph JJ, Aboagye-Mensah EB, Azap RA, Gray DM (2020). Community-engaged and community-based participatory research to promote American Heart Association Life's Simple Seven among African American adults: a systematic review. PLoS ONE.

[24] Amiri A, Zhao S (2019). Working with an environmental justice community: nurse observation, assessment, and intervention. Nurs Forum.

[25] Nash D, Magder L, Lustberg M, Sherwin RW, Rubin RJ, Kaufmann RB (2003). Blood lead, blood pressure, and hypertension in perimenopausal and postmenopausal women. JAMA.

[26] Victor RG, Lynch K, Li N, Blyler C, Muhammad E, Handler J (2018). A Cluster-randomized trial of blood-pressure reduction in Black barbershops. N Engl J Med.

[27] Schoenthaler AM, Lancaster KJ, Chaplin W, Butler M, Forsyth J, Ogedegbe G (2018). Cluster randomized clinical trial of FAITH (Faith-Based Approaches in the Treatment of Hypertension) in Blacks. Circ Cardiovasc Qual Outcomes.

[28] Balcazar HG, Byrd TL, Ortiz M, Tondapu SR, Chavez M (2009). A randomized community intervention to improve hypertension control among Mexican Americans: using the promotoras de Salud community outreach model. J Health Care Poor Underserved.

[29] Walters KL, LaMarr J, Levy RL, Pearson C, Maresca T, Mohammed SA (2012). Project həli?dx(w)/healthy hearts across generations: development and evaluation design of a tribally based cardiovascular disease prevention intervention for American Indian families. J Prim Prev.

[30] Felix HC, Narcisse M-R, Long CR, English E, Haggard-Duff L, Purvis RS (2019). The effect of family diabetes self-management education on self-care behaviors of Marshallese adults with type 2 diabetes. Am J Health Behav.

[31] Gonzalez MB, Herman KA, Walls ML (2020). Culture, social support, and diabetes empowerment among American Indian adults living with type 2 diabetes. Diabetes Spectr.

[32] McElfish PA, Rowland B, Riklon S, Aitaoto N, Sinclair KA, Ima S (2019). Development and evaluation of a blood glucose monitoring YouTube video for Marshallese patients using a community-based participatory research approach. Policy Polit Nurs Pract.

[33] Berkley-Patton J, Bowe Thompson C, Bauer AG, Berman M, Bradley-Ewing A, Goggin K (2020). Multilevel diabetes and CVD risk reduction intervention in African American churches: project faith influencing transformation (FIT) feasibility and outcomes. J Racial Ethn Health Disparities.

[34] Manjunath C, Jenkins SM, Phelan S, Breitkopf CR, Hayes SN, Cooper LA (2021). Association of body image dissatisfaction, behavioral responses for healthy eating, and cardiovascular health in African-American women with overweight or obesity: a preliminary study. American Journal of Preventive Cardiology.

[35] Derose KP, Williams MV, Branch CA, Flórez KR, Hawes-Dawson J, Mata MA (2019). A community-partnered approach to developing church-based interventions to reduce health disparities among African-Americans and Latinos. J Racial Ethn Health Disparities.

[36] Derose KP, Williams MV, Flórez KR, Griffin BA, Payán DD, Seelam R (2019). Eat, pray, move: a pilot cluster randomized controlled trial of a multilevel church-based intervention to address obesity among African Americans and Latinos. Am J Health Promot.

[37] Bess KD, Frerichs L, Young T, Corbie-Smith G, Dave G, Davis K (2019). Adaptation of an evidence-based cardiovascular health intervention for rural African Americans in the Southeast. Prog Community Health Partnersh.

[38] Ceasar JN, Claudel SE, Andrews MR, Tamura K, Mitchell V, Brooks AT (2019). Community engagement in the development of a mhealth-enabled physical activity and cardiovascular health intervention (step it up): pilot focus group study. JMIR Formativ Res.

[39] Brewer LC, Hayes SN, Jenkins SM, Lackore KA, Breitkopf CR, Cooper LA (2019). Improving cardiovascular health among African-Americans through mobile health: the FAITH! app pilot study. J Gen Intern Med.

[40] Brewer LC, Fortuna KL, Jones C, Walker R, Hayes SN, Patten CA (2020). Back to the future: achieving health equity through health informatics and digital health. JMIR Mhealth Uhealth.

[41] Moore QL, Kulesza C, Kimbro R, Flores D, Jackson F (2020). The role of prosocial behavior in promoting physical activity, as an indicator of resilience, in a low-income neighborhood. Behav Med.

[42] Paré ER, Body K, Gilsdorf S, Lucarelli J (2019). Qualitative focus groups: perceived influences on decision making about diet and physical activity among Hispanic/Latino participants. Health Promot Pract.

[43] Walters KL, Johnson-Jennings M, Stroud S, Rasmus S, Charles B, John S (2020). Growing from our roots: strategies for developing culturally grounded health promotion interventions in American Indian, Alaska Native, and Native Hawaiian communities. Prev Sci.

[44] Schultz K, Walters KL, Beltran R, Stroud S, Johnson-Jennings M (2016). “I’m stronger than I thought”: Native women reconnecting to body, health, and place. Health Place.

[45] Salvatore AL, Noonan CJ, Williams MB, Wetherill MS, Jacob T, Cannady TK (2019). Social support and physical activity among American Indians in Oklahoma: results from a community-based participatory research study. J Rural Health.

[46] Apata J, Sheikhattari P, Bleich L, Kamangar F, O’Keefe AM, Wagner FA (2019). Addressing tobacco use in underserved communities through a peer-facilitated smoking cessation program. J Community Health.

[47] Daley CM, Greiner KA, Nazir N, Daley SM, Solomon CL, Braiuca SL (2010). All nations breath of life: using community-based participatory research to address health disparities in cigarette smoking among American Indians. Ethn Dis.

[48] Patten CA (2012). Tobacco cessation intervention during pregnancy among Alaska Native women. J Cancer Educ.

[49] Lynch E, Emery-Tiburcio E, Dugan S, White FS, Thomason C, Jenkins L (2019). Results of ALIVE: a faith-based pilot intervention to improve diet among African American church members. Prog Community Health Partnersh.

[50] Mamun A, Kitzman H, Dodgen L (2020). Reducing metabolic syndrome through a community-based lifestyle intervention in African American women. Nutr Metab Cardiovasc Dis.

[51] Ralston PA, Wickrama KKAS, Coccia CC, Lemacks JL, Young-Clark IM, Ilich JZ (2020). Health for hearts united longitudinal trial: improving dietary behaviors in older African Americans. Am J Prev Med.

[52] Heredia NI, Lee M, Mitchell-Bennett L, Reininger BM (2017). Tu Salud ¡Sí Cuenta! Your health matters! A Community-wide Campaign in a Hispanic Border Community in Texas. J Nutr Educ Behav.

[53] Bird Jernigan VB, Salvatore AL, Williams M, Wetherill M, Taniguchi T, Jacob T (2019). A healthy retail intervention in Native American convenience stores: the THRIVE community-based participatory research study. Am J Public Health.

[54] Jernigan VBB, Wetherill M, Herod J, Jacob T, Salvatore AL, Cannady T (2017). Cardiovascular disease risk factors and health outcomes among American Indians in Oklahoma: the THRIVE study. J Racial Ethn Health Disparities.

[55] Smith I, White BM (2021). Barriers and facilitators to adhering to the American Heart Association’s Life’s Simple 7 for African American women living in public housing. J Health Care Poor Underserved.

[56] Joseph JJ, Nolan TS, Williams A, McKoy A, Zhao S, Aboagye-Mensah E (2022). Improving cardiovascular health in black men through a 24-week community-based team lifestyle change intervention: the Black impact pilot study. Am J Prev Cardiol.

[57] Brewer LC, Balls-Berry JE, Dean P, Lackore K, Jenkins S, Hayes SN (2017). Fostering African-American improvement in total health (FAITH!): an application of the American Heart Association’s Life’s Simple 7™ among Midwestern African-Americans. J Racial Ethn Health Disparities.

[58] Brewer LC, Morrison EJ, Balls-Berry JE, Dean P, Lackore K, Jenkins S (2019). Preventing cardiovascular disease: participant perspectives of the FAITH! program. J Health Psychol.

[59] Cacari-Stone L, Wallerstein N, Garcia AP, Minkler M (2014). The promise of community-based participatory research for health equity: a conceptual model for bridging evidence with policy. Am J Public Health.

[60] Kondo MC, South EC, Branas CC (2015). Nature-based strategies for improving urban health and safety. J Urban Health.

[61] South EC, MacDonald J, Reina V (2021). Association between structural housing repairs for low-income homeowners and neighborhood crime. JAMA Netw Open.

